# The Efficiency of Xylanase in Broiler Chickens Fed with Increasing Dietary Levels of Rye

**DOI:** 10.3390/ani9020046

**Published:** 2019-01-31

**Authors:** Anna Arczewska-Wlosek, Sylwester Swiatkiewicz, Dorota Bederska-Lojewska, Sylwia Orczewska-Dudek, Witold Szczurek, Danuta Boros, Anna Fras, Ewa Tomaszewska, Piotr Dobrowolski, Siemowit Muszynski, Małgorzata Kwiecien, Tomasz Schwarz

**Affiliations:** 1 Department of Nutrition Physiology, National Research Institute of Animal Production, 32-083 Balice, Poland; s.swiatkiewicz@izoo.krakow.pl (S.S.); dorota.bederska@izoo.krakow.pl (D.B.-L.); sylwia.orczewska@izoo.krakow.pl (S.O.-D.); witold.szczurek@izoo.krakow.pl (W.S.); 2 Laboratory of Quality Evaluation of Plant Materials, Institute of Plant Breeding and Acclimatization - National Research Institute, Radzikow, 05-870 Blonie, Poland; d.boros@ihar.edu.pl (D.B.); a.fras@ihar.edu.pl (A.F.); 3 Department of Animal Physiology, Faculty of Veterinary Medicine, University of Life Sciences in Lublin, 20-950 Lublin, Poland; ewaRST@interia.pl; 4 Department of Comparative Anatomy and Anthropology, Faculty of Biology and Biotechnology, Maria Curie-Skłodowska University, 20-033 Lublin, Poland; piotr.dobrowolski@umcs.lublin.pl; 5 Department of Physics, Faculty of Production Engineering, University of Life Sciences in Lublin, 20-950 Lublin, Poland; siemowit.muszynski@up.lublin.pl; 6 Department of Animal Nutrition and Feed Science, Institute of Animal Nutrition and Bromathology, Faculty of Biology, Animal Sciences and Bioeconomy, University of Life Sciences in Lublin, 20-950 Lublin, Poland; malgorzata.kwiecien@up.lublin.pl; 7 Department of Swine and Small Animal Breeding, Institute of Animal Science, Faculty of Animal Science, University of Agriculture, 30-059 Krakow, Poland; rzschwar@cyf-kr.edu.pl

**Keywords:** broiler chickens, rye, xylanase, growth performance, viscosity of small intestine content

## Abstract

**Simple Summary:**

In this study, the effect of xylanase addition to a diet with an increasing content of modern hybrid rye was evaluated. In the first feeding phase (from 1 to 21 days of age), even a low dietary level of rye (5%) decreased the body weight gain of birds. However, results obtained in older birds indicated that modern hybrid rye can be used in up to 20% dietary level in broiler diets from 22 to 42 days of age, especially when the diet is supplemented with xylanase.

**Abstract:**

In this paper, we present a study on the evaluation of the effect of xylanase addition to a diet with an increasing content of modern hybrid rye (Brasetto variety) on the performance indices and viscosity of small intestine content in broiler chickens. A total of 560 1-day-old male Ross 308 chickens were randomly assigned to 1 of 10 treatments, each comprising 7 replicate cages, with 8 male birds per cage. A 5 × 2 factorial arrangement was employed, with five dietary levels of ground rye (0%, 5%, 10%, 15%, and 20%). All the diets were either unsupplemented or supplemented with xylanase (200 mg/kg of feed; with minimum xylanase activity 1000 FXU/g). In the starter rearing period (1–21 days of age), the inclusion of rye (without xylanase supplementation) to the diet, even at the lowest dietary level (5%), negatively affected body weight gain (*p* < 0.05); there was no effect on feed intake and feed conversion ratio. In older chickens (the grower-finisher rearing period; 22–42 days of age), none of the dietary levels of rye (5–20%) affected growth performance indices. Similarly, no significant effect of increasing dietary level of rye was found for the entire rearing period (1–42 days of age). Diet supplementation with xylanase improved body weight gain and feed conversion ratio in chickens from 1 to 21 days of age. No positive effect of enzyme was found in older birds. No significant effects of the experimental factors used were noticed on the results of slaughter analysis, i.e., the carcass yield, breast meat yield, abdominal fat, and relative weight of the liver and gizzard. A high dietary concentration of rye (20%) increased the viscosity of small intestine content (*p* < 0.05); however, diet supplementation with xylanase significantly alleviated this effect. The findings of this experiment indicated that modern hybrid rye grain may be used at a 20% dietary level in broiler diets during the second feeding phase, i.e., from 22 to 42 days of age, without any detrimental influence on growth performance indices, while enzyme (xylanase) positively affected body weight gain and feed conversion ratio in younger chicks (1–21 days of age).

## 1. Introduction

The use of rye grain as a component of diets for poultry in conditions of intensive production is very limited. The reason for its reputation for being unsuitable for broiler chicken diets is its high concentration of nonstarch polysaccharides (NSP), mainly structural, highly branched arabinoxylans (AX), in the endosperm cell wall, which create viscous water solutions [1,2]. A substantial level of rye grain as an NSP source in the poultry diet can lead to enhanced digesta viscosity, slower digesta passage rate, and impaired absorption of nutrients which, in turn, depresses growth performance [3,4,5,6,7,8]. Another negative effect of increased digesta viscosity in chickens fed rye-based diets can be the excretion of sticky droppings [9,10]. Negative effects of high-NSP diets can be ameliorated by enzyme (xylanase) supplementation. Several previous experiments have shown that supplementation of rye-based diets with enzymes capable of hydrolyzing arabinoxylans can significantly reduce the degree of NSP polymerization and the viscosity of intestinal content, resulting in a decrease of the detrimental effects of high dietary AX concentration in poultry [4,7,11,12,13]. 

In recent years, several new hybrid rye varieties tending to have significantly reduced content of anti-nutritive substances, especially AX, have been developed [14,15,16] and have been become available on the market, being alternative, less expensive sources of energy and protein for poultry and livestock that can be used for the formulation of low-cost diets. In addition to their improved nutritional properties, new hybrid varieties of rye are also characterized by increased yield potential and resistance to fungus and pests, as well as reduced production costs [16]. Therefore, the objective of this experiment was to evaluate the influence of enzyme (xylanase) addition to diets with increasing levels (0%, 5%, 10%, 15%, 20%) of hybrid rye (Brasetto variety) on growth performance, and the results of slaughter analysis and digesta viscosity in broiler chickens. 

## 2. Materials and Methods

### 2.1. Birds and Experimental Diets

A total of 560, 1-d-old, male Ross 308 chickens were used. These were obtained from a commercial hatchery and had an average initial weight of 41 g. The birds were housed in wire-floored cages, in an environmentally controlled room in the poultry house at the Experimental Station of the National Research Institute of Animal Production in Balice, Poland. During the study, the temperature in the experimental facility was maintained from 32 °C at 1 d of age to 21 °C at 21 d of age and later, relative humidity cycled from 50 to 60%, air exchange was 1 m^3^/1 kg of BWG/1 h, and concentration of CO_2_ and NH_3_ were maintained below 2000 and 20 ppm, respectively. The chicks were weighed with BD3T electronic scales with an accuracy of 0.1 g (AXIS Sp. z o.o., Gdańsk, Poland), and randomly assigned to 1 of 10 treatments, each comprising 7 replicate cages, with 8 birds per cage (with 7800 cm^2^ total floor space in the cage). From 1 to 42 d of age, all the chickens were provided with water and feed ad libitum. 

All the birds were reared up to 42 d of age and fed with crumbled starter (1 to 21 d) and pelleted grower–finisher (22 to 42 d) isonitrogenous and isoenergetic diets, which were formulated to meet or exceed the nutrient requirements of broilers [17] (Table 1 and Table 2). Rye grain was ground using a 5-mm sieve size. A 5 × 2 factorial arrangement was employed, with five dietary levels of ground rye of Brasetto variety (0%, 5%, 10%, 15%, and 20%). All the diets were either unsupplemented or supplemented with xylanase (200 mg/kg of feed; Ronozyme WX, (CT)) with minimum xylanase activity 1000 FXU/g (DSM Nutritional Products Sp. z o.o., Mszczonów, Poland).

The nutrient content of the diets was calculated from the chemical composition of raw feedstuffs, and metabolizable energy value, in line with equations from the European Tables [18]. Air-dried samples of grains and experimental diets were analyzed for dry matter (DM), ash, crude protein (CP), and crude fat (CF) and acid detergent fiber (ADF) contents using standard analytical Association of Official Analytical Chemists (AOAC) procedures [19] (procedure numbers 934.01, 942.05, 954.01, 920.39 and 973.18, respectively). Neutral detergent fiber (NDF) was determined according to Van Soest et al. [20] using an Ankom^220^Fiber Analyser (Ankom Technology Corp., NY, USA) with heat-stable amylase and expressed inclusive of residual ash. The amino acid composition was determined by liquid chromatography (LC) using a AAA 400 amino acid analyzer (INGOS Co, Prague, Czech Republic) operating with an ion exchange column and post-column derivatization with ninhydrin.

NSP content with its fractionation to soluble (S-NSP) and insoluble (I-NSP) fractions was determined using gas chromatography (GC) as previously described by Englyst and Cummings [21]. In this procedure, the NSP of each fraction is a sum of individual monomers: Arabinose, xylose, mannose, galactose, and glucose. After enzymatic hydrolysis of starch, the samples were centrifuged and split into soluble (ethanol precipitates from supernatant) and insoluble (remaining pellet) fractions. Each of these fractions was hydrolyzed with 1M sulphuric acid (100 °C, 2 h) to monosaccharides and converted to volatile alditol acetates. The alditol acetates were separated on a capillary quartz column Rtx-225 (0.53 mm × 30 m) using the Clarus 500 gas chromatograph (Perkin Elmer) equipped with an autosampler, splitter injection port, and flame ionization detector. The carrier gas was He. Separation was performed at 225 °C, injection and detection at 275 °C.

Uronic acids (UA) were analyzed with the colorimetric method of Scott [22], using 3,5-dimethylphenwhich, which is highly reactive to UA derivatives. UA were calculated by measuring the difference between absorbance at 400 nm and 450 nm on a UV-1601 spectrophotometer (Beijing Rayleigh Analytical Instrument Co., Beijing, China). A solution of D-glucuronic and D-galacturonic acids at a 1:1 ratio was used as a standard.

The viscosity of water extracts was estimated with the modified method of Boros et al. [23]. The amount of total phenolic content (TPC) was determined according to the Folin–Ciocalteu procedure [24,25]. Absorption was measured at 765 nm (UV-1601, BRAIC). The total phenolic content was expressed as gallic acid equivalents (GAE) in milligrams per gram dry matter. 

### 2.2. Measurements

The chickens’ body weight was recorded at 1, 21, and 42 d of age; feed intake was recorded for 1–21- (starter diet) and 22-42-day (grower–finisher diet) periods, and mortality was registered daily. At 1 and 21 days of age, birds were weighed with BD3T electronic scales with an accuracy of 0.1 g (AXIS Sp. z o.o., Gdańsk, Poland), whereas at 42 days with Tru-Test AG-500 electronic scales with an accuracy of 5 g (Tru-Test Ltd, Auckland, New Zealand). The body weight gain (BWG) and feed conversion ratio (FCR) were calculated for the starter period (1–21 d), the finisher phase (22–42 d), and the entire feeding period (1–42 d of age). The mortality rate was recorded daily, and the weights of all mortalities were registered to correct the FCR. 

At the end of the experiment, and after 12 h of starvation, all of the chickens were individually weighed and 7 representative birds (one bird per each replicate cage) with live body weights close to the group average were chosen from each group, marked with number signs, and decapitated after electrical stunning (150 mA, frequency of 200 Hz for 4 s). Chickens were plucked, the intestines and crop were removed, and carcasses stored overnight at 4 °C. The mass of the cooled carcass with edible giblets (gizzard, liver) was estimated and carcass yield calculated. The breast muscles (pectoral major plus pectoral minor muscles), abdominal fat, livers, and gizzards were excised and weighed. The breast muscle and abdominal fat contents were expressed as a percentage of the cold carcass. The weights of the liver and gizzard were expressed as percentages of live weight [26]. 

Moreover, in selected treatments (diets without or with 20% of rye, not supplemented or supplemented with xylanase) for 7 non-fasting birds per treatment, the whole intestines were removed and total intestinal digesta from jejunum and ileum were collected for viscosity measurements. Fresh digesta were centrifuged at 10,000 × *g* for 10 min. Viscosity was measured on 0.5 mL of the supernatant using a cone and plate Brookfield viscometer (model DV2T LVCP, Brookfield Viscometers Ltd., Essex, UK) at 40 °C.

### 2.3. Statistical Analysis

The data were analyzed using STATISTICA 12 (StatSoft Inc., Tulsa, OK, USA) software for a two-way analysis of variance (ANOVA) to determine the main effects of dietary treatments, such as dietary rye levels and xylanase supplementation, and their interactions. Duncan’s multiple range post hoc test was used to determine the differences between the treatments, and the effects were considered to be significant at a probability level of *p* ≤ 0.05.

## 3. Results

The chemical composition of rye grain (Brasetto variety) used in our study included: 89.1% DM, 9.23% CP, 0.81% CF, 1.46% of crude fibre, and 1.51% of crude ash and apparent metabolizable energy—10.97 MJ/kg. The content of essential amino acids in the used rye variety was 1.19 g/kg for methionine (Met), 2.97 for lysine (Lys), 2.63 for threonine (Thr), 3.79 for valine (Val), and 2.50 for isoleucine (Ile). High soluble NSP content in rye grain resulted in its increased content in rye-containing diets and the high viscosity of water extracts (Table 2). 

Inclusion of ground rye, at all used dietary concentrations, except the level of 15%, significantly reduced BWG, without affecting feed intake (FI) and FCR in the starter rearing period (Table 3). No negative effects of dietary ground rye on performance indices were found during grower–finisher and entire rearing periods (Table 4 and Table 5).

Statistical analysis showed that the second experimental factor, i.e., dietary supplementation with xylanase, had a positive influence on BWG and FCR during the starter rearing period (Table 3). In older broilers during the grower–finisher period (Table 4), no effect of enzyme was found; however, xylanase improved FCR for the entire rearing period (Table 5). The significant interaction between experimental factors for BWG in the starter rearing period (xylanase addition improved BWG only in broilers fed rye-containing diet) was found (Table 3). 

The experimental factors used had no effect on the results of the slaughter analysis (*p* > 0.05). Thus, the carcass yield, breast meat yield, abdominal fat pad, and relative weight of the liver and gizzard were not affected by ground rye dietary level or enzyme addition to the diet (Table 6). 

The viscosity of intestinal jejunal and ileal digesta analyzed in selected treatments is presented in Table 7. Results of the analysis showed that high (20%) dietary levels of hybrid rye significantly increased the viscosity of small intestine (in jejunum and ileum) content, but enzyme addition reduced this disadvantageous effect of rye (*p* < 0.05). The significant interactions between experimental factors for viscosity values (the effect of xylanase on the jejunum and ileum content was much more pronounced in broilers fed rye-containing diet) were found (Table 7).

## 4. Discussion

The hybrid rye grain (Brasetto variety) used in this experiment was characterized by a relatively high concentration of both total and soluble NSP. Lower amounts of NSP in rye grain were found in harvest years 1997 [27] and 2010 [28]. The different content of the NSP in the rye grain may be the result of many factors, mainly genetics (rye varieties), climatic conditions, harvest date or the use of different analytical methods for the same feed material resulting in different data [27,29].

Analysis of the chemical composition of rye grain used in our experiment showed a relatively high content of NSP; however, the good growth performance of older chickens (22–42 days of age) fed the experimental diets indicated that hybrid rye can be used effectively as alternative feed material for broilers. Inclusion of rye grain, except the level of 15%, to the diet negatively affected growth performance only in younger broilers during the starter feeding phase (1–21 days of age), which might be connected with the high content of arabinoxylans in this rye; thus, a higher intestinal viscosity and greater susceptibility to its negative influence than was the case with older chickens was observed. According to Smulikowska et al. [30], in addition to the low digestibility of nutrients, and fat, especially, in rye-based diets, high digesta viscosity may also negatively affect the development of the stomach and the motility of the small intestines in young chickens. Older broilers, i.e., aged 22–42 days, were not as susceptible to the presence of NSP in the diet, so we did not find any negative effects of rye grain on performance during the grower–finisher rearing period. Findings of other authors regarding the effects of rye inclusion to the diet on broiler performance are consistent and frequently show a negative influence of high, i.e., 20% or more, dietary levels of rye on BWG and/or FCR. However, the magnitude of such adverse effects is not always the same, and this mainly depends on the chemical composition (NSP concentration) of the rye variety used. For instance, Lazaro et al. [31] reported that feeding broilers with a diet containing 50% rye negatively impaired growth performance indices (BWG and FCR), during both the first (to 25 days of age) and second (to 46 days) rearing periods. Mourão and Pinheiro [32] observed reduced BWG, worsened FCR, as well as decreased nutrient digestibility in chickens fed a diet containing 53% of rye. Józefiak et al. [33] found that performance indices of chickens fed a rye-based diet (62% of rye) were significantly worsened as compared to triticale- or wheat-based diets, which might be caused by the high concentrations of soluble arabinoxylans in rye-based diets. More recently, van Krimpen et al. [8] showed that even a relatively low dietary level of rye, i.e., 10%, reduced the growth performance of broilers. 

In the current study, xylanase addition significantly improved body weight and feed conversion ratios in younger age chickens, i.e., aged from 1 to 21 days, probably by improvements in nutrient use, which in younger chickens was affected to a greater extent by dietary inclusion of rye. Similar findings were obtained by Santos et al. [34], who concluded, based on growth performance results, that the action of xylanase used as supplement to rye-based diets for broiler chickens can be restricted to the first 21 days of birds’ life. Correspondingly, Mendes et al. [35] found a positive effect of xylanase addition on body weight gain, but not on feed intake or feed conversion ratio, in young broilers (1–28 days of age) fed a diet with a high level of triticale (hybrid of rye and wheat). Lazaro et al. [31] reported, however, that the growth performance of broilers fed a rye-based diet was improved by xylanase addition at all ages, i.e., during 1–25 d, as well as 26–46 days of age. Similarly, Silva and Smithard [36] observed that xylanase addition to a diet with 60% of rye significantly improved growth indices, which was attributed by the authors to the effect of enzyme on a decrease in the viscosity of intestinal fluid and increased nitrogen and fat digestibility. The lack of a significant influence of experimental factors (rye inclusion to the diet, xylanase addition) on results of slaughter analysis, among others carcass and breast meat yields, is in accordance with some previous studies [12,34,35]. However, Mourão and Pinheiro [32] reported decreased carcass yield in broilers fed a diet containing 53% of rye.

Although the viscosity values obtained in our experiment were generally relatively low compared to results of other experiments [36], the viscosity of intestinal content was significantly increased in chickens fed the diet containing the highest (20%) level of rye. This observation is in agreement with results of several previous studies aimed at an evaluation of the nutritional efficacy of rye in poultry diets. For instance, Tellez et al. [37] showed significantly increased gut viscosity in broilers fed a rye-based diet, which could be responsible for a decrease in growth performance indices and for alteration in the intestinal microbiota composition and bone mineralization. The same adverse effects of high dietary level of rye have been observed in turkey poults [38]. Lee et al. [39] found that a 40% inclusion rate of rye to broiler diets significantly enhanced the viscosity of jejunum and ileum digesta, which was associated with reduced body weight gains, feed conversion ratios and fat digestibility.

We observed that supplementation of a diet containing 20% of rye with xylanase had a positive effect on intestinal physiology and decreased digesta viscosity. Similarly, Dänicke et al. [40] found that xylanase addition to diets with a high level of rye (61%) significantly reduced the viscosity of intestinal fluid in broilers aged 14 or 28 days. Further, similar results on the influence of xylanase supplementation on intestinal viscosity in chickens fed rye-based diets were reported by Józefiak et al. [33] and Lazaro et al. [31]. Xylanase also reduced digesta (duodenum + jejunum) viscosity in the case of the use of triticale-based diets [35]. 

## 5. Conclusions

The results of this experiment showed that hybrid rye (Brasetto variety) can be incorporated in up to 20% of the diet without any detrimental effects on the growth performance of broilers from 22 days of age. However, inclusion of hybrid rye to the diets of younger chickens, aged 1–21 days, may reduce body weight gains and worsen feed conversion ratios. The positive effect of enzyme (xylanase) addition to diets containing rye was reflected in a decrease in the viscosity of small intestine contents.

## Figures and Tables

**Table 1:** Composition and nutrient content of experimental diets, g/kg air dry matter.

Item	Starter (1–21 d)					Grower-Finisher (22–42 d)				
	Control	Rye-Containing Diets				Control	Rye-Containing Diets			
Rye	0	50	100	150	200	0	50	100	150	200
Corn	457.1	405.1	354.1	319.1	302.1	404.4	353.4	298.4	263.4	238.4
Wheat	100	100	100	80	40	200	200	200	180	150
Soybean meal	370	368	365	366	370	306	304	304	305	307
Rapeseed oil	33	37	41	45	48	52	55	60	64	67
Limestone	13.5	13.5	13.5	13.5	13.5	14	14	14	14	14
Monocalcium phosphate	15	15	15	15	15	13	13	13	13	13
NaCl	3	3	3	3	3	3	3	3	3	3
DL-Methionine	2.6	2.6	2.6	2.6	2.6	2.3	2.3	2.3	2.3	2.3
L-Lysine HCl	0.8	0.8	0.8	0.8	0.8	1.7	1.7	1.7	1.7	1.7
L-Treonine	-	-	-	-	-	0.6	0.6	0.6	0.6	0.6
Vitamin–mineral premix ^1^	5	5	5	5	5	3	3	3	3	3
Calculated composition:										
Metabolizable energy, MJ/kg ^2^	12.6					13.1				
Crude protein	225					205				
Lys	12.3					11.5				
Met	5.8					5.25				
Thr	8.5					8.1				
Ca	9.7					9.3				
Total P	7.1					6.6				
Available P	4.5					4.1				
Acid detergent fiber (ADF)	99.3	108.9	103.2	107.6	104.7	106.4	103.3	103.5	109.0	109.5
Neutral detergent fiber (NDF)	50.4	52.2	47.9	49.5	51.0	49.5	49.5	48.2	51.3	51.4

^1^ The premix provided, per 1 kg of starter diet: Vitamin A (retinol), 3.75 mg; vitamin D_3_ (cholecalciferol), 1.25 mg; vitamin E (alpha-tocopherol), 125 mg; vitamin K_3_ (menadione), 3 mg; vitamin B_1_ (thiamine), 3 mg; vitamin B_2_ (riboflavin), 8 mg; vitamin B_6_ (pyridoxine), 4 mg; vitamin B_12_ (cyanocobalamin), 0.02 mg; biotin, 0.2 mg; Ca-pantotenate, 16,3 mg; niacin, 50 mg; folic acid, 2 mg; choline chloride, 348 mg; manganese, 100 mg; zinc, 100 mg; iron, 50 mg; copper, 20 mg; iodine, 1 mg; selenium, 0.35 mg; coccidiostat: 100 ppm of total narasin/nicarbazin activity; per 1 kg of finisher diet: Vitamin A (retinol), 3.0 mg; vitamin D_3_ (cholecalciferol), 0.75 mg; vitamin E (alpha-tocopherol), 30 mg; vitamin K_3_ (menadione), 2.5 mg; vitamin B_1_ (thiamine), 2.5 mg; vitamin B_2_ (riboflavin), 5 mg; vitamin B_6_ (pyridoxine), 3.51 mg; vitamin B_12_ (cyanocobalamin), 0.021 mg; biotin, 0.201 mg; Ca-pantotenate, 13 mg; niacin, 35 mg; folic acid, 1.0 mg; choline chloride, 300 mg; manganese, 80 mg; zinc, 90 mg; iron, 40 mg; copper, 20 mg; iodine, 0.5 mg; selenium, 0.2 mg; coccidiostat:narasin, 70 ppm. ^2^ Calcuated according to the European Table [18] as a sum of the ME content of components.

**Table 2:** Chemical composition and viscosity of water extracts of the experimental diets and rye grain.

Item	Diets					Rye Grain
	Rye 0%	Rye 5%	Rye 10%	Rye 15%	Rye 20%	
	**Starter feeding phase (1–21 d)**					
Soluble NSP (g/kg DM)	16.9	15.4	17.2	19.7	18.0	55.0
Insoluble NSP (g/kg DM)	92.6	85.2	75.1	79.5	93.9	100
Total NSP (g/kg DM)	109.5	100.6	92.3	99.1	111.9	155
Arabinoxylans (g/kg DM)	37.9	35.7	36.0	36.5	41.4	85.5
Simple sugars (g/kg DM)						
Arabinose	18.7	17.7	17.9	18.6	20.2	30.2
Xylose	19.1	18.0	18.1	17.9	21.2	55.4
Mannose	8.20	7.79	6.67	7.69	7.60	6.73
Galactose	19.8	17.3	16.2	18.7	20.4	3.95
Glucose	41.3	37.7	31.7	33.9	40.0	58.7
Uronic acids (g/kg DM)	14.6	13.3	12.8	13.5	13.5	3.01
Total phenolic content (mg GAE/g DM)	26.7	25.3	25.3	25.5	26.1	12.2
Viscosity of water extract (mPa × s)	10.6	12.3	15.0	17.4	18.3	9.47
	**Finisher feeding phase (22–42 d)**					
Soluble NSP (g/kg DM)	12.8	12.5	15.6	16.8	19.7	
Insoluble NSP (g/kg DM)	92.6	94.7	95.3	91.8	97.9	
Total NSP (g/kg DM)	105.4	107.2	110.9	108.6	117.6	
Arabinoxylans (g/kg DM)	41.1	40.5	41.9	42.9	46.1	
Simple sugars (g/kg DM)						
Arabinose	19.9	20.0	20.6	20.9	22.0	
Xylose	21.2	20.5	21.3	22.0	24.1	
Mannose	7.05	7.22	7.57	7.46	7.63	
Galactose	17.3	19.1	18.3	18.2	19.0	
Glucose	37.7	37.8	40.5	37.6	42.5	
Uronic acids (g/kg DM)	9.90	9.20	9.40	10.60	9.10	
Total phenolic content (mg GAE/g DM)	25.1	25.1	24.4	23.3	23.9	
Viscosity of water extract (mPa × s)	11.0	11.3	13.8	15.2	18.5	

NSP: Nonstarch polysaccharides; DM: Dry matter; GAE: Gallic acid equivalents.

**Table 3:** Effects of dietary treatments on broiler performance in starter period of feeding ^1^, 1–21 d of age.

Treatments	Body Weight Gain (g)	Feed Intake (g)	Feed Conversion Ratio (g/g)
**Rye dietary level (%):**			
0	965 ^a^	1280	1.33
5	933 ^b^	1256	1.35
10	936 ^b^	1256	1.34
15	949 ^ab^	1268	1.34
20	928 ^b^	1247	1.35
**Xylanase supplementation:**			
No	930 ^a^	1271	1.37 ^a^
Yes	954 ^b^	1252	1.31 ^b^
**SEM**	4.25	6.39	0.007
***p*-value**			
Rye	0.007	0.497	0.807
Xylanase	0.001	0.134	0.0001
Interaction	0.007	0.208	0.208

Means of seven replicates of eight chickens kept in one cage. ^a, b^—the values with different letters differ significantly (*p* ≤ 0.05).

**Table 4:** Effects of dietary treatments on broiler performance in grower/finisher period of feeding ^1^, 22–42 d of age.

Treatments	Body Weight Gain (g)	Feed Intake (g)	Feed Conversion Ratio (g/g)
**Rye dietary level (%):**			
0	1950	3240	1.66
5	1956	3248	1.66
10	1959	3248	1.66
15	1950	3246	1.66
20	1925	3223	1.68
**Xylanase supplementation:**			
No	1947	3240	1.66
Yes	1949	3242	1.66
**SEM**	9.88	17.0	0.06
***p*-value**			
Rye	0.864	0.991	0.948
Xylanase	0.935	0.952	0.984
Interaction	0.991	0.972	0.872

Means of seven replicates of eight chickens kept in one cage.

**Table 5:** Effects of dietary treatments on broiler performance in entire period of feeding ^1^, 1–42 d of age.

Treatments	Body Weight Gain (g)	Feed Intake (g)	Feed Conversion Ratio (g/g)
**Rye dietary level (%):**			
0	2914	4509	1.55
5	2889	4503	1.56
10	2895	4499	1.55
15	2899	4510	1.56
20	2853	4467	1.57
**Xylanase supplementation:**			
No	2877	4509	1.57 ^a^
Yes	2903	4486	1.55 ^b^
**SEM**	10.9	17.0	0.06
***p*-value**			
Rye	0.503	0.944	0.692
Xylanase	0.261	0.537	0.015
Interaction	0.724	0.727	0.855

Means of seven replicates of eight chickens kept in one cage. ^a, b^—the values with different letters differ significantly (*p* ≤ 0.05).

**Table 6:** Effects of dietary treatments on results of slaughter analysis.

Treatments	Carcass Yield (%)	Breast Meat Yield (% of Carcass)	Abdominal Fat (% of Carcass)	Relative Weight of Liver (% of Live Weight)	Relative Weight of Gizzard (% of Live Weight)
**Rye dietary level (%):**					
0	78.7	31.5	1.15	1.74	0.485
5	78.9	31.4	1.29	1.74	0.519
10	78.8	31.1	1.16	1.68	0.549
15	79.2	30.5	1.22	1.68	0.499
20	78.9	31.4	1.12	1.68	0.515
**Xylanase supplementation:**					
No	78.9	31.0	1.21	1.71	0.515
Yes	78.9	31.3	1.16	1.70	0.512
**SEM**	0.128	0.155	0.030	0.017	0.009
***p*-value**					
Rye	0.727	0.277	0.387	0.598	0.189
Xylanase	0.969	0.308	0.433	0.806	0.888
Interaction	0.276	0.216	0.115	0.918	0.123

Means of seven replicates (of seven chickens).

**Table 7:** Viscosity of the intestinal contents.

Treatments	Viscosity of the Jejunum Contents (mPa × s)	Viscosity of the Ileum Contents (mPa × s)
**Rye dietary level (%):**		
0	1.56 ^a^	1.56 ^a^
20	3.26 ^b^	3.21 ^b^
**Xylanase supplementation:**		
No	3.02 ^b^	3.36 ^b^
Yes	1.80 ^a^	1.41 ^a^
**SEM**	0.226	0.273
***p*-value**		
Rye	<0.0001	<0.0001
Xylanase	<0.0001	<0.0001
Interaction	<0.0001	<0.0001

Means of seven replicates (of seven chickens). ^a, b^—the values with different letters differ significantly (*p* ≤ 0.05).
